# Predictability of the psychological impact of the economic crisis on family functioning within the family context in Sri Lanka

**DOI:** 10.3389/fpsyg.2025.1587710

**Published:** 2025-10-01

**Authors:** Sasini Uditha, Asanka Bulathwatta

**Affiliations:** Department of Psychology, University of Peradeniya, Peradeniya, Sri Lanka

**Keywords:** economic crisis, family functioning, family resilience, psychological well-being, gross domestic product (GDP)

## Abstract

**Introduction:**

The financial crisis due to COVID-19 and other socio-political constraints led to family vulnerability, leading to psychological disorders that disrupt individual well-being and family dynamics and relationships. Researchers emphasize the importance of predicting and mitigating these psychological effects to avoid long-term adverse outcomes. The main objectives were to understand the factors contributing to family resilience and the psychological impact of financial hardship on family functioning in the Sri Lankan context.

**Methods:**

This study examined whether the economic crisis impacts family functioning within the family context. The participants received a questionnaire comprising four subsections to collect quantitative data. The first section was for informed consent, and the second was for demographic-related questions. Section three was about Walsh family resilience, 32 scales. The fourth section comprised self-structured questions. Participants were asked to answer all these questions. The research study targets families in the Mavathalandha Grama Niladhari Division, Rathnapura district, Sri Lanka. Sample size of 100 families were selected using a stratified sampling method. The Statistical Package for Social Sciences version 22 was used to analyze and interpret data.

**Results:**

The study’s regression analysis results helped identify some resilience factors that directly and indirectly impact the economic crisis on family functioning within the family context. Also, in the comparative analysis of studies on family resilience, the research findings revealed the factors contributing to family resilience and the psychological impact of financial hardship on family functioning within the family context during the economic crisis.

**Discussion:**

These findings highlight the significance of family belief systems, positive communication, and adaptive problem-solving as key components of family resilience during economic and psychological stress to provide insights into effective strategies for supporting family well-being during times of economic hardship.

## Introduction

The economic crisis that has affected Sri Lanka since 2019, exacerbated by the COVID-19 pandemic and other socio-political challenges, has significantly intensified the country’s financial instability (World Bank, 2022). In this dire context, various mental health disorders have emerged, disrupting individual well-being, family dynamics, and relationships among family members (Trulik et al., 2025). The crisis has also led to rising unemployment and a sharp increase in the cost of living, further deepening financial insecurity (Asian Development Bank, 2022a,b). As a result, many Sri Lankan families have struggled to meet their basic needs. These unexpected and prolonged stressors have triggered considerable emotional distress, anxiety, and other mental health concerns among family members (World Bank, 2022), further straining family relationships and roles (Walsh, 2015). Therefore, examining the factors that contribute to family resilience and mental well-being during economic crises is essential. Despite these challenges, some families demonstrate resilience, allowing them to manage the effects of economic stress more effectively (Luthar et al., 2000). Family resilience refers to the ability to adapt to adversity, maintain strong emotional bonds, and communicate effectively (Walsh, 2016a,b). Positive strategies, such as open communication, effective problem-solving, seeking social support, and maintaining a hopeful outlook, are vital for fostering resilience and sustaining psychological well-being (Walsh, 2015). The emotional well-being of family members plays a central role in overall family functioning (Walsh, 2015). However, elevated levels of stress and anxiety can disrupt individual roles within the family, weaken relationships, and increase conflict (Walsh, 2003). Finally, Sri Lanka’s ongoing economic crisis has had a profound impact on the psychological well-being of families and has disrupted family dynamics. Nevertheless, family resilience can serve as a protective factor, enabling families to maintain stability and well-being during periods of crisis. Understanding the factors that influence family resilience is especially important within the Sri Lankan context.

### Research background

#### Economic crisis and psychological well-being during the economic crisis

Several factors have contributed to the decline in psychological well-being during economic crises. Rising unemployment rates, reduced income, increasing living expenses, and uncertainty about the future have disrupted daily life and financial stability (World Health Organization, 2021a,b). Psychological well-being comprises multiple dimensions, including personal growth, purpose in life, positive relationships with others, environmental mastery, self-acceptance, and autonomy (Ryff, 1989).

During times of economic crisis, these pressures can overwhelm individuals’ coping mechanisms. According to Lazarus and Folkman (1984), economic stress can lead to feelings of hopelessness, low self-esteem, and heightened vulnerability to mental health issues. Moreover, financial difficulties often impact family dynamics, exacerbating conflicts, weakening family cohesion, and straining relationships. These disruptions can reduce emotional support systems and negatively affect the overall family environment, potentially leading to long-term adverse psychological outcomes (Walsh, 2015).

#### Family functioning and resilience within the family context during economic crisis

Economic crises significantly impact family dynamics, often resulting in increased stress, depression, and various mental health disorders. These crises can profoundly alter family roles and relationships. Emotional distance between family members and financial pressures can disrupt daily routines, weaken familial bonds, and impair effective communication. Core family values such as cooperation, mutual support, and harmonious living are often undermined. These disruptions can lead to heightened conflict, increased stress levels, decreased emotional intimacy, strained marital relationships, and a higher risk of divorce (Conger and Elder, 1994). The emotional and psychological toll on families facing prolonged financial instability is especially severe. Family members may experience high levels of stress, anxiety, and depression. Children raised in such environments are also at risk of developing emotional and behavioral problems, which further compromise overall family functioning (McCubbin and Patterson, 2014a,b).

**Figure 1:**
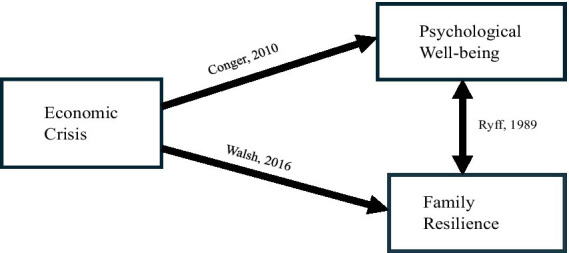
Conceptual framework.

Family resilience refers to a family’s ability to adapt, recover, and thrive during times of economic hardship. Resilient families can maintain strong relationships, cope effectively with stress, and adapt to adversity. Strategies that enhance resilience include developing new skills, seeking alternative sources of income, fostering open communication, engaging in mutual support, and practicing effective problem-solving (Walsh, 2003). Social support is also crucial in strengthening family resilience and helping families navigate economic challenges (Walsh, 2015). Moreover, maintaining a positive outlook and setting realistic expectations can enhance a family’s ability to cope with mental and emotional strain during economic downturns. Comparative studies on resilience have identified both protective and risk factors. According to Masten and Powell (2003) strong familial relationships, effective communication, and optimism are key protective factors that foster resilience. Conversely, high stress levels, poor communication, and limited social support can hinder resilience. These findings suggest that while some families possess inherent resilience traits, others may benefit from external interventions aimed at enhancing their coping mechanisms. Theoretical frameworks such as Family Resilience Theory and tools like the Walsh Family Resilience Questionnaire (WFRQ) offer valuable insights into how families manage adversity. They underscore the importance of adaptation, flexibility, and resourcefulness in building resilience and ensuring family stability during economic crises (Figure 1).

### Conceptual framework

#### Aim

This research study aims to investigate the predictability of the psychological impact of the economic crisis on Sri Lankan family functioning, with a special focus on the interaction of family resilience and family dynamics.

#### Objectives

One major goal is to identify the main psychological stressors connected with the economic crisis, assess their influence on various aspects of family functioning, and investigate the role of family resilience as a moderating factor in reducing negative consequences. By understanding these relationships, the research seeks to investigate the psychological impact of financial hardship on family functioning in the context of economic crisis and the factors that contribute to family resilience during economic difficulties.

#### Research questions

Research Question 1: Is there any impact of gender on individual well–being within the family context during an economic crisis?

Research Question 2: Is there any impact of income level on individual well-being within the family context during an economic crisis?

Research Question 3: Is there any impact of family communication and problem-solving processes on individual well-being within the family context during an economic crisis?

Research Question 4: Is there any impact of family belief system within the family context on individual well-being within the family context during an economic crisis?

#### Hypothesis

H1: There is a significant impact of gender on individual well–being within the family context.

H2: Dual-salary earners experience greater individual psychological well-being within the family context.

H3: Better communication and problem-solving are linked to higher well-being.

H4: The family belief system is associated with higher psychological well-being.

## Materials and methods

This study employed a quantitative survey research design to investigate the psychological impact of the ongoing economic crisis on family functioning in Sri Lanka. The primary objective was to examine the predictability of this impact by assessing levels of family resilience and psychological well-being using standardized and structured instruments.

Participants were recruited using stratified sampling from households within the Mavathalandha Grama Niladhari Division in the Ratnapura District. The sample comprised adults aged 18 years and above who were literate in either Sinhala or English. Households were selected based on official community records provided by the local Grama Niladhari officer, ensuring diverse representation. Participation was entirely voluntary, and all individuals were informed about the purpose of the study, the confidentiality of their responses, and their right to withdraw at any time without consequence. Before data collection, written informed consent was obtained from all participants.

Data were gathered using a structured questionnaire distributed in printed form. To ensure linguistic and conceptual clarity, the questionnaire was provided in both Sinhala and English, following a process of translation and back-translation to preserve semantic accuracy.

The questionnaire consisted of four sections. The first section was the Informed Consent Form, which provided a clear explanation of the study’s aims, the voluntary nature of participation, confidentiality assurances, and instructions for withdrawal. Participants indicated their consent by signing this section. The second section captured demographic information, including variables such as age, gender, marital status, number of children, type of employment, monthly income, and educational level.

The third section assessed family resilience using the Walsh Family Resilience Questionnaire (WFRQ), developed by Walsh (2015). This instrument comprised 32 items across three key domains: Family Belief Systems (such as making meaning of adversity, maintaining a positive outlook, and spirituality), Organizational Patterns (including family flexibility, connectedness, and mobilizing external resources), and Communication and Problem-Solving (such as emotional expression, clear communication, and collaborative decision-making). Items were rated on a 5-point Likert scale, ranging from 1 (strongly agree) to 5 (strongly disagree). WFRQ demonstrated high internal consistency in this study, indicating strong psychometric reliability.

The fourth and final section, here these six questions were informed by existing research on GDP and economic stress indicators and grounded in Carol Ryff’s six aspects of psychological well-being (Ryff, 1989) each representing one of Ryff’s six dimensions: autonomy, environmental mastery, personal growth, positive relations with others, purpose in life, and self-acceptance. Responses were also recorded on a 5-point Likert scale, same as WFRQ. The scale demonstrated acceptable reliability.

### Family resilience questionnaire (WFRQ)

In this study, data were collected using the Family Resilience Questionnaire (WFRQ), developed by Walsh (2015). The questionnaire comprises 32 items designed to assess various dimensions of family resilience. It is structured into three main sections: the family belief system, family organizational processes, and communication and problem-solving processes. Each of these sections is further subdivided into key components. The family belief system includes making meaning of adversity, a positive outlook, and transcendence/spirituality. The family organizational processes section encompasses flexibility, connectedness, and mobilizing social and economic resources. The communication and problem-solving section include clear and consistent messages, open emotional expression, and collaborative problem-solving. The questionnaire uses a 5-point Likert scale ranging from 1 (strongly agree) to 5 (strongly disagree) and typically takes about 10 min to complete. In its initial validation, the WFRQ demonstrated excellent internal consistency, with alpha reliability estimates above (*α* = 0.952).

### Psychological well-being questionnaire according to Carol Ryff’s six subscales

This section comprises self-structured questions designed to measure the psychological and functional impacts of the economic crisis on individuals within the family context. These six questions were informed by existing research on GDP and economic stress indicators and grounded in Carol Ryff’s six aspects of psychological well-being, providing a nuanced understanding of the interplay between economic stressors and family dynamics. This questionnaire is on a Likert scale from 1 strongly agree to 5 strongly disagree. This takes about 5 min to complete. Alpha reliability estimates above (*α* = 0.981).

### Sample

This study was conducted with a total of 100 families, selected using a stratified sampling technique to ensure proportional representation across key demographic categories within the target population. Stratification was based on gender, ethnicity, and socioeconomic status, utilizing demographic data obtained from the Grama Niladhari officer’s community records. Each stratum was proportionally represented in the final sample to enhance representativeness and reduce potential sampling bias. Within each stratum, families were selected systematically according to a pre-established list to ensure an unbiased and organized selection process.

To be eligible for inclusion in the study, families were required to be permanent residents of the Mavathalandha Grama Niladhari Division in the Ratnapura District, Sri Lanka. Additionally, each participating family had to include at least one married adult aged 18 years or older, and all participants were required to provide informed consent and demonstrate a willingness to engage in the survey process.

Exclusion criteria were also established to maintain the integrity and quality of the data. Families were excluded if they experienced significant language barriers, and no translation assistance was available. Furthermore, families who declined participation or were unreachable after three documented contact attempts were omitted from the study. In addition, families that included members with cognitive or physical disabilities that hindered their ability to complete the questionnaire independently were not included in the final sample.

This stratified sampling approach was designed to ensure that each family within the defined population had an equal probability of being selected, also preserving the demographic diversity necessary for producing generalizable and valid research findings.

### Data analysis

Data for this study were collected through a structured questionnaire administered to 100 selected families. Once the data collection process was completed, all responses were systematically entered and analyzed using IBM SPSS Statistics software. The analysis involved three primary statistical techniques. Simple linear regression analysis, Correlation analysis and additionally, Analysis of Variance (ANOVA). These analytical methods provided a comprehensive understanding of the Psychological Impact of the economic crisis on Family Functioning within the Family Context in Sri Lanka.

### Ethical considerations

This study was conducted by the ethical guidelines established by the American Psychological Association (APA). Ethical approval was obtained before data collection, ensuring that all procedures adhered to established standards for the protection of participants’ rights and welfare.

Permission to conduct the study was formally obtained from the Grama Niladhari officer of the Mavathalandha Grama Niladhari Division, who also provided an official list of families residing in the area. Participants were then selected using a stratified sampling method based on this list, ensuring representation across key demographic characteristics such as gender, ethnicity, and socioeconomic status.

All participants were provided with detailed information about the purpose of the study, the procedures involved, and any potential risks. Written informed consent was obtained only after participants confirmed they understood their voluntary involvement and their right to withdraw from the study at any time without penalty.

Strict measures were taken to uphold confidentiality and anonymity throughout the research process. No personally identifiable information was recorded or disclosed. All data were securely stored and used solely for academic purposes, and responses were analyzed and presented in aggregate form to ensure participant privacy, particular attention was paid to minimizing any potential psychological harm. The questionnaire was carefully designed using sensitive and inclusive language, and participants had the option to skip any questions they found distressing or uncomfortable. Participation was entirely voluntary, with no form of coercion applied during recruitment. By adhering to the APA’s ethical standards and obtaining local administrative approval, this study ensured that all research activities were conducted with integrity, respect, and due consideration for the dignity and well-being of participants.

## Study results

The research sample consists of a 100 participants, and the gender distribution indicates a slight female predominance with 55% of respondents being female and 45% being male. The majority (35%) fall within the 30–40 age range. This is followed by 25% in the 40–50 age group, 22% in the 50–60 range, and 18% aged 60 or older. In terms of household income structure, 44% of participants belong to single-wage earner households, whereas 56% are from dual-wage earner families. Among 37% earn Rs. 80,000 and Rs. 95,000 per month and 33% of respondents make less than Rs. 35,000 per month. On the other hand, those earnings between Rs. 35,000 to Rs. 50,000 are 9% and Rs. 50,000 to Rs. 65,000 are 2% and Rs. 65,000 to Rs. 80,000 are 3%, respectively. Meanwhile, 16% of respondents report earning more than Rs. 95,000. All participants in this study are married, with family sizes varying based on the number of children, since there are 100 respondents, the percentages correspond directly to these frequencies, with 4% having no children, 19% having one child, 23% having two children, 29% having three children, and 25% having more than three children. A correlation analysis was conducted. So, the correlations between several variables were examined. The key variables are gender, income type, family belief system, family communication and problem-solving process, and Carol Ryff’s six aspects of psychological well-being.

Hypothesis one: Psychological well-being, measured by using Carol Ryff’s six dimensions, had a mean score of 18.43 and a standard deviation of 8.84. According to the Pearson correlation coefficient, the relationship between gender and psychological well-being was −0.024, indicating no statistically significant relationship (*p* = 0.405). This implies that there is no significant relationship between gender and psychological well-being, with a very weak negative association. And simple linear regression was conducted to examine the impact of gender on psychological well-being. Gender explains less than 0.1% of the variance in psychological well-being, according to the R-squared value of 0.001. Moreover, according to one-way ANOVA analysis, the F-statistics are 0.058 with a *p*-value of 0.810, indicating that the regression model is not statistically significant. These results indicate that gender does not have a significant impact on psychological well-being within the family context during the economic crisis. So, Hypothesis One was not accepted.

Hypothesis two: Here, the objective is to investigate whether there is any impact of income level on individual well-being within the family context. To test this hypothesis, Regression analysis, Pearson correlation analysis, and one-way ANOVA were conducted. Based on the descriptive statistics, the mean score for psychological well-being measured by Carol Ryff’s six aspects of psychological well-being is 18.43 with a standard deviation of 8.84. The mean score for income type, which indicates whether the family has a single salary or a dual salary, is 1.56 with a standard deviation of 0.49. Moreover, the correlation analysis revealed a strong positive correlation (*R* = 0.91) between income type (single vs. dual salary) and psychological well-being, which is statistically significant (*p* < 0.001). This indicates that families with dual salaries have better psychological well-being. Moreover, regression analysis was used to investigate how psychological well-being is impacted by the type of income (single vs. dual salary) with an *R*-squared value of 0.840, indicating that approximately 84% of the variance in psychological well-being can be explained by income type. Furthermore, the regression model is statistically significant; also, ANOVA analysis results indicate the regression model is statistically significant [*F*(1.98) = 513.986, *p* < 0.001]. These results show that income type has a significant effect on psychological well-being. The strong positive correlation and significant regression coefficients suggest that families with dual-wage earners experience greater psychological well-being. Therefore, these research findings support hypothesis two.

Hypothesis three: The objective of this analysis is to determine whether there is any impact of family communication and problem-solving processes on individual well-being during the economic crisis. As the same as the above hypothesis, regression analysis, Pearson correlation analysis, and one-way ANOVA were conducted. The dependent variable was the sum of scores from Carol Ryff’s six aspects of psychological well-being questionnaire, and the independent variable was the sum of scores from the communication and problem-solving processes subsection in WFRQ. The R-value of 0.549 indicates a moderate correlation between communication and problem-solving processes and psychological well-being. An *R* Square value of 0.302 suggests that approximately 30% of the variance in psychological well-being can be explained by family communication and problem-solving processes. The ANOVA results show that the regression model is statistically significant, with an *F*-value of 42.389 and a significance level of <0.001. The analysis shows that psychological well-being during an economic crisis is significantly impacted by family communication and problem-solving processes. The moderate correlation and significant regression results indicate that better communication and problem-solving in the family context are associated with higher psychological well-being. Therefore, the results support hypothesis three, which states that the process of family communication and problem-solving during the economic crisis has an impact on individual well-being.

Hypothesis Four: This is to investigate any impact of family belief systems within the family context on individual well-being. To test this hypothesis, linear regression analysis, Pearson correlation analysis, and one-way ANOVA were conducted. To check the impact of family belief systems on individual well-being, a regression analysis was conducted using sum scores of Carol Ryff’s six aspects of psychological well-being as the dependent variable. The independent variable was the sum score of the family belief system sum score in the subsection of WFRQ. The correlation coefficient (*R*) is 0.638, indicating a moderate positive relationship between family belief systems and individual well-being. The *R*-squared value of 0.407 suggests that approximately 41% of the variance in individual well-being can be explained by family belief systems. Furthermore, the regression model’s overall significance was evaluated using ANOVA. The *F*-value is 67.301, and the *p*-value is <0.001, indicating that the regression model is statistically significant. The analysis shows that family belief systems significantly impact psychological well-being during economic crises. The moderate correlation and significant regression results suggest that better family belief systems within the family context are associated with higher individual psychological well-being. Hence, the results support hypothesis 04, which says that there is an impact of the family belief system on individual well-being during the economic crisis (Tables 1–5).

**Table 1:** Mean and SD among background information.

	Mean	St. deviation	Number
Gender	1.55	0.500	100
Income type	1.56	0.499	100
SUM; Family belief system	29.0000	8.81688	100
SUM; Family organizational process	22.6500	7.04298	100
SUM; Communication and problem-solving process	21.0500	6.77246	100
SUM; Carol Ryff’s Six Aspects of Psychological Well-being	18.4300	8.83548	100

**Table 2:** Regression summary table.

Variables in regression	*R*	*R* ^2^	*F* Statistics	*P*-value
Gender	0.024	0.001	0.058	0.810
Income type	0.916	0.840	513.986	<0.001
Communication and problem-solving process	0.549	0.302	42.389	<0.001
Family belief system	0.638	0.407	67.301	<0.001

**Table 3:** Correlation summary table.

Variable 1	Variable 2	Pearson correlation	Significant
Gender	Carol Ryff’s six psychological Well-being	−0.405	>0.001
Income type	Carol Ryff’s six psychological Well-being	0.916	<0.001
Communication and problem-solving process	Carol Ryff’s six psychological Well-being	0.549	<0.001
Family belief system	Carol Ryff’s six psychological Well-being	0.638	<0.001

**Table 4:** Linear regression summary table.

Hypothesis	Dependent variable	Independent variable	*R*	*R* ^2^	*F* statistics	*p*-value	Significance Yes/No
(1) There is a significant impact of gender on individual well–being within the family context	Individual well–being within the family context	Gender	0.024	0.001	0.058	0.810	No
(2) Dual salary earners experience greater individual psychological well-being within the family context.	Individual psychological well-being within the family context	Income type	0.916	0.840	513.986	<0.001	Yes
(3) Better communication and problem-solving linked to higher well-being	Individual well being (Carol Ryff’s six aspects of psychological well-being)	Communication and problem-solving sub-section in the Family Resilience Questionnaire (WFRQ)	0.549	0.302	42.389	<0.001	Yes
(4) The family belief system is associated with higher psychological well-being	Individual well-being (Carol Ryff’s six aspects of psychological well-being)	Family belief system sub-section in the Family Resilience Questionnaire (WFRQ)	0.638	0.407	67.301	<0.001	Yes

**Table 5:** Correlations summary table.

	Gender	Income type	Family belief system	Family organizational process	Communication and problem-solving process	Carol Ruff’s six aspects of psychological well-being
Gender	1					
Income type	0.089	1				
Family belief system	−0.550**	−0.551**	1			
Family organizational processes	−0.401**	−0.682**	0.944**	1		
Communication and problem-solving process	−0.661**	−0.588**	0.901**	0.876**	1	
Carol Rff’s six aspects of psychological well-being	−0.024	0.916**	−0.638**	−0.767**	−0.549**	1

**Correlation is significant at the 0.01 level (2-tailed).

## Discussion

The discussion section interprets the findings about existing literature, offering key insights into psychological well-being within the family context during an economic crisis. This study explores how variables such as gender, income level, family communication and problem-solving strategies, and family belief systems contribute to psychological resilience amidst financial instability. By integrating these findings with prior research, this section aims to provide a comprehensive understanding of the complexities of psychological well-being in families facing economic challenges.

According to this study, gender does not significantly impact individual psychological well-being within the family context during an economic crisis in Sri Lanka. While previous literature often emphasizes the influence of gender on well-being in such contexts, some studies suggest that the impact of gender may be limited or context dependent. Research focusing on access to psychosocial resources, such as social support and coping strategies, indicates that these factors can overshadow gender differences. When both men and women have equitable access to such resources, the influence of gender on psychological well-being may diminish (Umberson et al., 2010). The findings of this study suggest that contextual factors such as strong communal support, effective communication, and problem-solving capacities may mitigate gender disparities in psychological well-being. In cultures or families that promote egalitarian values and emphasize resilience and community support, gender differences tend to be less pronounced (Lyons et al., 1998). Additionally (Walsh, 2015) highlighted that families with strong resilience and coping abilities are more likely to maintain psychological well-being during economic downturns, shifting the focus to the resilience of the family unit rather than individual, gender-based experiences.

Income level was found to significantly influence psychological well-being, with dual-income households exhibiting greater resilience during periods of economic crisis. The financial stability provided by multiple income sources not only alleviates economic stress but also enhances a family’s perceived security and cohesion, contributing to overall mental health (Voydanoff, 2005a,b). Dual-earner households often demonstrate more adaptive coping mechanisms, as the shared economic responsibilities can buffer the psychological impact of financial strain (Moen et al., 2012) Additionally, research by Karney et al. (2005) and Mistry et al. (2002) emphasizes how consistent income sources contribute to more stable family dynamics and better emotional regulation during crises.

Effective family communication and problem-solving mechanisms were found to significantly enhance individual well-being during economic downturns. Families that engaged in open, supportive, and adaptive communication tended to display greater emotional resilience, cohesion, and lower levels of intra-family conflict (Walsh, 2012). Constructive problem-solving approaches, characterized by joint decision-making and emotional validation, allow family members to manage stress collaboratively, thereby reducing psychological distress and feelings of helplessness (Patterson, 2002a,b; Prime et al., 2020). Research has shown that such relational processes play a buffering role against the adverse effects of economic strain, promoting psychological stability and overall family functioning (Conger and Conger, 2002; Masten and Narayan, 2012). Furthermore, evidence highlights that effective communication and supportive family relationships foster resilience and contribute to positive outcomes for both children and families, underscoring their protective function in maintaining individual and collective well-being during economic hardship (Ministry of Social Development, 2003).

Furthermore, family belief systems comprising shared values, cultural traditions, and religious or spiritual practices have been consistently identified as vital components in fostering psychological well-being and resilience, particularly during times of crisis. According to Walsh (2015) strong belief systems provide families with a sense of coherence, emotional support, and meaning, which are essential in navigating adversity. These systems often reinforce collective identity and facilitate adaptive coping mechanisms by promoting hope, optimism, and a future-oriented outlook (Koenig, 2012). In the face of economic hardship, families with strong belief systems are more likely to exhibit resilience through meaning-making processes and spiritual coping strategies, which buffer against psychological distress (Park, 2013; Masten and Motti-Stefanidi, 2020). Such belief frameworks serve as protective factors by fostering intra-family solidarity and emotional regulation, ultimately reducing the negative mental health impacts associated with financial stress (Walsh, 2016a,b). These findings underscore the importance of integrating culturally and spiritually sensitive approaches into mental health interventions aimed at supporting families during economic downturns. Incorporating belief-based frameworks into mental health programs may enhance engagement, relevance, and outcomes for diverse populations facing systemic and financial adversity (Zábó et al., 2025).

Finally, this study highlights the multifaceted nature of psychological well-being within the family context during economic crises. It encompasses key components of family resilience, including gender, socioeconomic status, communication, problem-solving strategies, and belief systems. Future interventions should focus on enhancing family communication, strengthening problem-solving skills, and integrating belief systems to promote psychological stability. These findings are instrumental in guiding the development of culturally sensitive policies and therapeutic interventions designed to support families during economic hardship.

## Conclusion

This study emphasizes an intricate aspect of psychological well-being in a family context during an economic crisis in Sri Lanka. The findings contribute to a more nuanced understanding of family resilience through an investigation of gender, income levels, family communication and problem-solving, and family belief systems. Future interventions should emphasize enhancing family communication, developing adaptive problem-solving skills, and leveraging belief systems to assist families throughout economic downturns. These insights are crucial in designing culturally relevant policies and therapy treatments to help families function during difficult times.

### Limitations and suggestions for future researchers

This study’s main limitation is the relatively small sample size of 100 participants from the Mavathalandha Grama Niladhari Division, Rathnapura district, Sri Lanka which may limit the generalizability of the findings. Future research should use larger, more diverse samples across different regions to enhance external validity.

Despite these limitations, this study helps address a significant gap in Sri Lankan research. While the psychological impact of economic crises on families has been widely studied internationally, limited attention has been given to this issue in the Sri Lankan context. And the country’s unique socio-cultural and economic dynamics, further studies are essential to deepen understanding and develop culturally relevant interventions.

## Data Availability

The original contributions presented in the study are included in the article/supplementary material, further inquiries can be directed to the corresponding author/s.
