# Supplementary material for: Predictability of the psychological impact of the economic crisis on family functioning within the family context in Sri Lanka
Source: Front Psychol. 2025 Oct 1;16:1587710. doi: 10.3389/fpsyg.2025.1587710 (PMC12520910; doi:10.3389/fpsyg.2025.1587710)
Supplement: Supplementary file 1 [file Supplementary_file_1.pdf]

APPENDIX

Consent Form

Dear participant,

I am Sasini Uditha from the University of Peradeniya, a candidate for honors in psychology. For my final year Dissertation Research Project done under the supervision of Dr. Asanka Bulathwatta, I'm conducting a study on "Predictability of the psychological impact of the economic crisis on family functioning within the family context in Sri Lanka " The questionnaire will require 15 minutes to answer, and all the inputs will be used for research purposes only. Throughout the study, I vow to maintain the confidentiality and anonymity of the participants. You will not face any harm, disadvantage, or direct or indirect benefit from this research. You are free to withdraw from this study at any time. You are allowed to ask for clarification and ask questions from the researcher about the study or about the procedure at any time.

Sasini Uditha (Researcher)

Department of Psychology,

Faculty of Arts,

University of Peradeniya,

0702401423

Udithasasini@gmail.com

Dr. Asanka Bulathwatta

(Lecturer),

Department of Psychology,

Faculty of Arts,

University of Peradeniya,

Asankabulathwatta@gmail.com

Thank you!

I have understood from the information given that I possess the opportunity to ask questions. I understand that my participation is voluntary and that I am free to withdraw at any time. I agree to participate in this research voluntarily.

Yes

No

Questionnaire:

1) Age

Below 30 years

30<= age <40

40<= age <50

50<= age <60

Above 60 years

2) Gender

Male

Female

3) Family Income Type

A single salary

A dual salary

4) Family monthly income

Below: 35,000 RS

35,000 <= RS < 50,000

50,000 <= RS < 60,000

65,000 <= RS < 80,000

80,000 <= RS < 95,000

Above: RS: 95,000

5) Marital status

Married

Single

6) Number of children

None

One

Two

Three

More than three

7) Education level

O/L, A/L

Bachelor’s Degree

Master's Degree

PhD

Other...

Family Questionnaire (WFRQ)

	Strongly disagree	Disagree	Neutral	Strongly agree	Agree
1) We face adversities together, not alone.	5	4	3	2	1
2) We see the distress that accompanies our condition as common and understandable	5	4	3	2	1
3) We approach the crisis as a challenge that can be managed and mastered with collective efforts.	5	4	3	2	1
4) We try to understand our stressful situation and focus on our options/ alternatives.	5	4	3	2	1
5)We believe that we can overcome our difficulties.	5	4	3	2	1

	Strongly disagree	Disagree	Neutral	Strongly agree	Agree
6) We motivate each other and rise with our strength.	5	4	3	2	1
7) We make use of opportunities, act accordingly, and live in persistence.	5	4	3	2	1
8) We focus on our capabilities and accept the things that we can't change.	5	4	3	2	1
9) We share important values and goals that help us overcome adversity.	5	4	3	2	1
10) We engage spiritually (religious or non-religious) to face things more confidently.	5	4	3	2	1

	Strongly disagree	Disagree	Neutral	Strongly agree	Agree
11)Our challenge creativity create more meaningful priorities and stronger bonds.	5	4	3	2	1
12)Our hardships increased our companionship and desire to help others	5	4	3	2	1
13) We learn from our challenges and become stronger	5	4	3	2	1
14) We will become creative to adapt ourselves to new challenges.	5	4	3	2	1
15) We provide our family members with stability and reliability when they are under stress.	5	4	3	2	1

	Strongly disagree	Disagree	Neutral	Strongly agree	Agree
16)The resilient leadership of parent caregivers provides warm nurturing, guidance, and protection	5	4	3	2	1
17) We can count on our family members to help each other during hardships.	5	4	3	2	1
18)Our family respects our interests and differences	5	4	3	2	1
19) We have optimists and good advisors in our immediate and extended family.	5	4	3	2	1
20) We can place trust in our friends and our community.	5	4	3	2	1
21)We have the economic security to prevent difficulties	5	4	3	2	1

	Strongly disagree	Disagree	Neutral	Strongly agree	Agree
22) We can depend on community resources during hard times	5	4	3	2	1
23) We will try to explain the details of our stressful situation and alternatives.	5	4	3	2	1
24) We are direct and clear in what we say and do.	5	4	3	2	1
25)We can express our respective opinions and be honest with each other	5	4	3	2	1
26) We can share our difficult or negative emotions (Ex: sadness, anger, fear)	5	4	3	2	1
27)We understand each other without blaming others	5	4	3	2	1

	Strongly disagree	Disagree	Neutral	Strongly agree	Agree
28)We can share our positive feelings, appreciation, humor, and joy, and can find relief from hardships	5	4	3	2	1
29) We are collaborative when we discuss, make decisions, and handle disagreements fairly.	5	4	3	2	1
30) We focus and work towards our goals	5	4	3	2	1
31)We celebrate victory as well as learn from our mistakes.	5	4	3	2	1
32) We plan and prepare for our future to prevent crises or difficulties.	5	4	3	2	1

Carol Ryff's questionnaire is based on six aspects of Psychological Well-Being.

	Strongly Disagree	Disagree	Neutral	Strongly agree	Agree
1)During the economic crisis, to what extent did you feel that changes in your income influenced your ability to maintain control over your happiness and well-being?	5	4	3	2	1
2) Do you feel confident in your psychological ability to effectively manage and adapt to changes in your environment?	5	4	3	2	1
3) Since the onset of the economic crisis, have you experienced personal growth by adapting or improving your communication patterns within your family	5	4	3	2	1
4)Have you observed that the economic crisis impacted on the quality of relationships within your family and the nature of your family's social interactions and community involvement?	5	4	3	2	1
5) Have you observed any changes that affected your sense of purpose and overall Mental well-being during the economic crisis?	5	4	3	2	1
6)How has the social perception of the economic crisis influenced your sense of self-worth and acceptance of yourself?	5	4	3	2	1
